# ESF-EMBO Symposium “Molecular Biology and Innovative Therapies in Sarcomas of Childhood and Adolescence” Sept 29–Oct 4, Polonia Castle Pultusk, Poland

**DOI:** 10.3389/fonc.2013.00142

**Published:** 2013-06-04

**Authors:** Beat W. Schäfer, Ewa Koscielniak, Heinrich Kovar, Simone Fulda

**Affiliations:** ^1^Department of Oncology and Children’s Research Center, University Children’s Hospital Zurich, Zurich, Switzerland; ^2^Olgahospital, Klinikum Stuttgart, Stuttgart, Germany; ^3^Children’s Cancer Research Institute, St Anna Kinderkrebsforschung, Vienna, Austria; ^4^Institute for Experimental Cancer Research in Pediatrics, Goethe-University Frankfurt, Frankfurt, Germany

**Keywords:** Rhabdomyosarcoma, Ewing sarcoma, therapeutics, PAX3/FOXO1, EWS/Fli1

## Abstract

Rhabdomyosarcoma (RMS) and Ewing sarcoma (ES) are among the most common pediatric sarcomas (Arndt et al., 2012). Despite sarcomas representing a highly heterogeneous group of tumors, ES and alveolar RMS (ARMS) typically share one common genetic characteristic, namely a specific chromosomal translocation (Helman and Meltzer, 2003; Lessnick and Ladanyi, 2012). These translocations generate fusion proteins, which are composed of two transcription factors (TF). Typically, one TF is a developmentally regulated factor that is essential for proper specification of a given lineage and provides the DNA-binding domain, while the partner TF contributes a transactivation domain that drives aberrant expression of target genes. Based on these common genetic characteristics, the first ESF-EMBO research conference entitled “Molecular Biology and Innovative Therapies in Sarcomas of Childhood and Adolescence” with special focus on RMS and ES was held at the Polonia Castle in Pultusk, Poland. The conference gathered 70 participants from more than 15 countries and several continents representing most research groups that are active in this field.

Alveolar RMS (ARMS) harbors reciprocal chromosomal translocations between chromosome 2 (or chromosome 1) and chromosome 13, yielding *t*(2;13), or *t*(1;13). These translocations lead to expression of characteristic fusion proteins that consist of the DNA-binding domain of PAX3 or PAX7 and the transactivation domain of the forkhead transcription factor FOXO1 on chromosome 13. Ewing sarcoma (ES) on the other hand is characterized by a translocation involving the ubiquitously expressed, putative RNA-binding EWSR1 gene and the friend leukemia integration 1 (FLI1) transcription factor, forming the EWS/FLI1 fusion protein as the most common fusion event.

The genetic basis of these diseases is therefore thought to be transcriptional deregulation of normal developmental processes. Hence, improving current treatment approaches will require likely a more detailed understanding of the biology of these oncogenic fusion proteins as well as of the underlying biology of the tumor cells. Importantly, however, available mouse models for ARMS using PAX3/FOXO1 as oncogenic driver as well as (so far unsuccessful) attempts to generate a mouse model for ES based on expression of EWS/FLI1 have unraveled the importance of largely unknown secondary hits which are required for tumorigenesis. Identification of such secondary hits likely requires large-scale next-generation sequencing of which several projects are now getting under way. One critical challenge in this area, however, is the availability of sufficient and high-quality tumor and normal tissue specimen.

Since transcription factors (TF) are thought to be undruggable based on their overall structure and the complexity of eukaryotic transcription, novel treatment approaches likely have to be indirect. One such indirect possibility might lie in the epigenetic control of gene expression which seems to play a major role also in sarcomas, as discussed during the conference. Additional areas that are beginning to emerge are the detailed characterization of the role played by embryonal signaling pathways such as Wnt, Notch, and Hedgehog (Hh) in tumorigenesis of sarcomas.

## Tumor Biology of Sarcoma

Different levels of gene regulation by EWS/FLI1 were discussed by H. Kovar (Vienna, Austria) who also investigated the role of the Notch pathway in ES. O. Delattre (Paris, France) described their attempts to generate a mouse model for ES and also described identification of an additional novel translocation, BCOR-CCNB3. The next two talks under this topic looked at specific aspects in rhabdomyosarcoma (RMS), namely the biological role of CB1, a well-known downstream target gene. Interestingly, inhibition of CB1 was found to abrogate lung metastasis in a xenograft mouse model (G. Grosveld, Memphis, TN, USA). Finally, S. Hettmer (Boston, MA, USA) reported on a promising *in vitro* system that allows transformation of primary satellite cells by specific oncogenes such as *RAS* together with deletion of *CDKN2A*, thereby creating a defined RMS tumorigenicity model.

## Transcriptional Control and Epigenetics

S. Lessnik (Salt Lake City, UT, USA) discussed a novel target gene repressed by EWS/FLI1, namely lysyl oxidase (LOX) that inhibits the transformed phenotype of ES cells. For transcriptional repression, association of EWS/FLI1 with histone deacetylases and the NuRD complex were identified as necessary elements. As a component of the Wnt signaling pathway, DKK2 was reported by G. Richter (Munich, Germany) to be highly overexpressed in ES and critical for malignant cell outgrowth *in vitro* and *in vivo*.

Apart from histone acetylation, also histone methylation is an important epigenetic mechanism contributing to genetic heterogeneity. E. Lawlor (MI, USA) described studies investigating the role of the polycomb repressor complex in response to EWS/FLI1 expression. Finally, activity of the fusion TF might also be regulated by protein–protein interactions. One interesting co-factor to follow up in the future might be E2F (R. Schwentner, Vienna, Austria).

## Identification of Novel Critical Pathways in Sarcoma Biology

T. Triche (Los Angeles, CA, USA) discussed the prognostic significance of non-coding RNA identified by a human exon array. In ES, ErbB4 might play a role in metastasis (P. Sorensen, Vancouver, BC, Canada), while cul4, an E3-ubiquitin ligase, might have a general role in ES (C. Mackintosh, Salamanca, Spain). In RMS, novel pathways that were subject of discussion included the role of pro-protein convertases which are involved in processing of important therapeutic targets such as IGF1R (M. Bernasconi, Zurich, Switzerland). Several novel target genes of PAX3/FOXO1 were characterized by different groups such as carnitine palmitoyltransferase and pleiotrophin (T. Chen, Memphis, TN, USA), JARID2 (J. Shipley, Sutton, UK), and p-cadherin (C. Gauthier-Rouviere, Montpellier, France). Genome-wide ChIP-seq experiments could also help to identify novel critical pathways. Such experiments were presented by L. Cao (Washington, DC, USA) for PAX3/FOXO1. Among the important receptor targets that he identified as direct targets of the fusion protein were FGFR4, c-MET, IGFR1R, and TRAIL receptor 2. Interestingly, about 25% of the identified binding sites seem to lie in the first intron and some even further downstream within genes. Another important new field for sarcoma biology is the role of miRNAs in the tumorigenic process. Some miRNA termed the myomiRs are important for myogenic differentiation and hence RMS biology. These include miR206 and miR1 which were discussed by S. Subramanian (Minneapolis, MN, USA) and C. Ponzetto (Torino, Italy).

## Resistance Mechanisms and Cancer Stem Cells

Despite its relevance for tumor biology, characterization of cancer stem cells in sarcoma is still in its infancy. Attempts to define a subpopulation of ES cells by side-population analysis were discussed by M. Hotfilder (Muenster, Germany). Evasion of apoptosis, one of the hallmarks of human cancers, can also be the cause of treatment resistance. Indeed, sensitivity of sarcoma cells to treatment with TRAIL was reported by two researchers (S. Fulda, Frankfurt, Germany and F. Redini, Nantes, France). Along similar lines, blockade of survivin might enhance sensitivity to therapeutic T cells (K. Simon-Keller, Mannheim, Germany). P. Zammit (London, UK) described an *in vitro* culture system of isolated single muscle fibers that contained satellite cells in their natural niche. Ectopic expression of PAX3/FOXO1 was able to induce expression of myogenin but not myoD. However, it remained unclear whether the satellite cells become fully transformed. D. Loeb (Baltimore, MD, USA) then looked at another interesting gene in the context of ES, namely WT1. This transcription factor might play an important role for angiogenesis in this tumor.

## Animal Models of RMS

An important tool not only to advance our understanding of tumor biology but also to prioritize drugs for pre-clinical development is the availability of genetically engineered animal models. While no mouse model is available up to now for ES that is driven by the EWS/FLI1 oncogene, C. Keller (Portland, OR, USA) described mice expressing PAX3/FOXO1 that will form ARMS in collaboration with p53 deletion. Interestingly, he used different driver genes along the myogenic lineage to initiate expression of the fusion protein and found the most penetrating model to be with myf6. From this data he concluded that the cell of origin for ARMS is a fetal myoblast. In contrast to ARMS, several mouse models are available that give rise to ERMS, the RMS subtype without expression of the fusion protein. H. Hahn (Goettingen, Germany) reported on recent findings in the Ptch heterozygous mouse model that leads to activation of the Hh pathway and ERMS. An interesting zebrafish model utilizing activated *K-RAS* was discussed by D. Langenau (Boston, MA, USA). This model allows *in vivo* imaging of developing tumors. While not all cells in a tumor might have tumor-propagating potential, these cells clearly can enter the vasculature and play a critical role in metastasis, at least in zebrafish.

## Fusion Proteins as Therapeutic Targets

J. Toretsky (Washington, DC, USA) discussed interaction of EWS/FLI1 with RNA helicase A which could be utilized as therapeutic target and the improvements that were made in developing a specific small therapeutic molecule. Another approach was described by B. Schäfer (Zurich, Switzerland) who investigated post-translational modifications of PAX3/FOXO1. Several phosphorylation sites were identified and a candidate upstream kinase (PLK1) was identified that might regulate PAX3/FOXO1 activity. The same phosphorylation sites were also investigated biochemically in much more detail by A. Hollenbach (New Orleans, LA, USA). Finally, the role of gene amplification in the pathogenesis of RMS was addressed by F. Barr (Bethesda, MD, USA).

## Targeting Signal Transduction

Several important signaling pathways were highlighted in this session. These included a CRKL/Yes axis (L. Helman, Bethesda, MD, USA), targeting of c-Met by a small-molecule inhibitor (S.J. Ding, NE, USA) and the influence of the Notch pathway on invasion of RMS cells (J. Roma, Barcelona, Spain). The possibility of targeting both tumor and stromal cells by inhibiting the PDGFR pathway was brought up by M. Ehman (Stockholm, Sweden). A new and exciting area of research is represented by the newly discovered ALK mutations that occur in RMS and which can be targeted by small-molecule inhibitors (Y. Versleijen-Jonkers, Nijmegen, Netherlands).

## New Drugs – Pre-Clinical and Clinical Evaluation

The last two sessions of the conference closed the circle to discuss potentially novel treatment options. The first talk in this session was given by P. Houghton (Columbus, OH, USA) who summarized the efforts made by the Pediatric Pre-clinical Testing Program (PPTP) to identify important novel drugs for sarcoma treatment. Interestingly, response rates for known drugs in the xenograft models are around 30%, while it was only 9% for experimental drugs. For sarcomas, one important class of drugs might be topoisomerase I inhibitors. Furthermore, inhibition of the IGF1R resulted in suppression of sarcoma growth. Since the PPTP program works with animal models, it will be crucial to develop strategies for clinical translation. J. Chisholm (Sutton, UK) described the structure used in Europe to achieve this goal, and which is called ITCC (innovative treatments for children with cancer). Several trials are now implemented under this umbrella. One of the targets that are currently being developed at the Royal Marsden was discussed in the final talk by S. Gatz (Sutton, UK), namely FGFR, which was found mutated in some ARMS and overexpressed in most of them.

## Clinical Research

Moving further to the clinics, U. Dirksen (Muenster, Germany) summarized the current state of the art on diagnosis, treatment and prognostic factors for ES in Europe. E. Koscielniak (Stuttgart, Germany) gave an overview on the role of molecular signatures in soft tissue sarcoma and their impact on therapy stratification of RMS. S. Stegmaier (Stuttgart, Germany) discussed the prognostic relevance of fusion transcript markers in soft tissue sarcoma. Some discrepancy in the field concerns the clinical outcome of different PAX/FOXO1 fusions, as different outcomes depending on distinct PAX/FOXO1 fusion products have been found in the US, but not in the European CWS trials. Finally, a novel fusion transcription factor, EWSR1/NFAT2c was described in ES (K. Szuhai, Leiden, Netherlands).

## Conclusion

This conference was the first specialized international meeting that brought together leading biologists and clinicians involved in early clinical trials to discuss recent advances in translational cancer research on two groups of sarcomas, namely RMS and ES, in the pediatric and young adult population. Since fusion TF are often the only genetic abnormality present at high frequency in these tumors, they are hypothesized to be one of the tumor-initiating events and might represent important therapeutic targets. Therefore, a large effort is still directed at identification of specific, either activated or repressed target genes. Several speakers discussed novel techniques such as next-generation sequencing and whole genome ChIP-seq to identify such target genes including mechanisms of transcriptional regulation. Several new targets genes that have now been individually characterized were highlighted as novel therapeutic targets at the conference. Another important area in this field is the development of genetic animal models to further advance our understanding of the biology of the diseases and to pre-clinically evaluate novel targeted compounds. A specific session addressed the progress in this field spanning from mouse to zebrafish models. Especially for ES, the lack of an appropriate mouse model still represents a major bottleneck.

In two forward-looking sessions, future research directions were discussed. Among them were methods to unravel the still unknown cell of origin for these sarcomas. Novel insights into this aspect might be provided by future collaborations with developmental biologists. Additional identified areas included the search for novel genetic alterations, the role of the micro- and macroenvironment, the significance of tumor heterogeneity and pharmacogenomics. Regarding novel therapeutic targets, more emphasis might be put on understanding the biology of the prevalent genetic aberrations found in these tumors, namely the fusion TF themselves. However, important downstream target genes include some well-known oncogenes against which direct inhibitors are available and therefore development of therapies using these targets might be more straightforward. Hopefully, a next conference planned 2 years from now will further prioritize the most promising therapeutic avenues.

## Conflict of Interest Statement

The authors declare that the research was conducted in the absence of any commercial or financial relationships that could be construed as a potential conflict of interest.
